# Bridging B-cell autoimmunity and oncology: the PD-1/PD-L1 paradox in systemic lupus erythematosus

**DOI:** 10.3389/fimmu.2026.1813842

**Published:** 2026-05-20

**Authors:** Yunfeng Guan, Can Chen, Yulong Hou

**Affiliations:** 1 Huzhou Central Hospital, Fifth School of Clinical Medicine of Zhejiang Chinese Medical University, Huzhou, China; 2 Huzhou Central Hospital, Affiliated Central Hospital of Huzhou University, Huzhou, China

**Keywords:** B cell immunotolerance, checkpoint agonists, immunotherapy, PD-1/PD-L1 signaling axis, systemic lupus erythematosus (SLE)

## Abstract

Systemic lupus erythematosus (SLE) remains one of the most challenging autoimmune diseases due to its complex pathogenesis involving the breakdown of B cell tolerance. While the PD-1/PD-L1 axis is traditionally viewed as a universal inhibitory checkpoint, its role in SLE is uniquely nuanced. In this review, we synthesize recent findings to clarify the “PD-1 Paradox”—the observation that high PD-1 expression on pathogenic T follicular helper (Tfh) and T peripheral helper (Tph) cells in SLE patients correlates with chronic activation and extrafollicular expansion rather than functional exhaustion. We also summarize emerging precision immunotherapy strategies, including PD-1 agonists, bispecific inhibitors (targeting ICOSL/BAFF), and engineered mesenchymal stem cells (MSCs). These interventions aim to move beyond traditional immunosuppression toward a precision “reset” of the autoimmune system.

## 1 Introduction

Systemic lupus erythematosus (SLE) is a prototypical systemic autoimmune disease characterized by a profound loss of tolerance to nuclear self-antigens, the production of pathogenic autoantibodies, and immune-mediated damage to multiple organ systems (1–3). The pathogenesis of SLE is complex and heterogeneous, involving intricate interactions between genetic susceptibility, environmental triggers, and widespread dysregulation of both innate and adaptive immunity (1, 2). At the heart of this dysregulation lies a fundamental breakdown in the checks and balances that normally maintain self-tolerance, leading to polyclonal B cell hyperreactivity and the sustained production of autoreactive antibodies (4, 5). These pathogenic autoantibodies, notably anti-double-stranded DNA (dsDNA) antibodies, are central effectors of tissue inflammation and organ damage, particularly in the kidneys and skin (1, 3). The mechanisms driving the generation and persistence of these autoreactive B cell clones are multifaceted, involving both T cell-dependent germinal center (GC) reactions and alternative extrafollicular activation pathways (6–8).

Within this context of immune hyperactivity and failed regulation, the programmed death-1 (PD-1)/programmed death-ligand 1 (PD-L1) axis emerges as a critical but paradoxical player. Originally identified as a pivotal immune checkpoint that limits T cell responses and maintains peripheral tolerance, the PD-1 pathway’s role in autoimmunity was unequivocally demonstrated by the spontaneous development of lupus-like glomerulonephritis and arthritis in PD-1-deficient mice (9). This foundational finding positioned PD-1 signaling as a non-redundant brake on autoreactive lymphocytes. However, the narrative in human SLE is nuanced and appears contradictory. While genetic ablation leads to autoimmunity, the expression and function of the PD-1/PD-L1 pathway within the dysfunctional immune milieu of established SLE are complex and dysregulated (10, 11). The axis is modulated by key SLE-related pathways, including Toll-like receptor (TLR) and type I interferon (IFN) signaling, which are notoriously overactive in patients (10, 12). This creates a scenario where a pathway essential for preventing autoimmunity is itself subject to the inflammatory disturbances it is supposed to constrain.

This paradox is particularly evident in the realm of B cell biology, which is central to SLE pathogenesis. B cells in SLE are not merely passive antibody producers; they are intrinsically hyper-reactive, serve as antigen-presenting cells (APCs), secrete pro-inflammatory cytokines, and form ectopic lymphoid structures (4, 5). Recent studies have highlighted the prominence of specific pathogenic B cell subsets, such as double-negative (IgD^-^CD27^-^) B cells, which exhibit a pre-plasma cell, extrafollicular phenotype, hyper-responsiveness to TLR7 signaling, and strong associations with active disease and nephritis (12). The generation of these autoreactive effector B cells and antibody-secreting cells can occur via the rapid EF pathway, a process potently fueled by type I IFN and plasmacytoid dendritic cells (pDCs) (8). The PD-1/PD-L1 checkpoint is intimately involved in regulating the T cell help essential for both GC and EF pathways, yet its net effect in SLE seems to be a failure to adequately restrain pathogenic humoral immunity. This failure may stem from cell-type-specific dysregulation, where inhibitory signals are insufficient on some lymphocyte subsets while promoting aberrant activation or dysfunction on others.

Therefore, the PD-1/PD-L1 axis in SLE represents a double-edged sword: it is a guardian whose loss precipitates disease, yet its presence within the inflamed environment is subverted, contributing to the very humoral autoimmunity it should suppress. This review will delve into this central paradox. We will explore the fundamental biology of the PD-1 pathway and its role in tolerance, examine its complex impact on T cell subsets that help B cells, and dissect its dual role in B cell biology. We will further consider contributions from innate immune and stromal cells, correlate axis dynamics with clinical disease, and evaluate emerging therapeutic strategies that aim to recalibrate this critical checkpoint. Resolving this paradox is essential for advancing toward precision immunotherapy in SLE, moving beyond broad immunosuppression to targeted restoration of immune homeostasis (2, 10, 11).

## 2 Fundamental biology of the PD-1/PD-L1 pathway and its role in immunological tolerance

The paradoxical role of the PD-1/PD-L1 axis in systemic autoimmunity is deeply rooted in its fundamental design as a critical immune checkpoint. This inhibitory pathway, central to maintaining self-tolerance, was elucidated through seminal discoveries linking its disruption to spontaneous autoimmune pathologies. The PD-1 receptor, a member of the immunoglobulin superfamily containing an immunoreceptor tyrosine-based inhibitory motif (ITIM) in its cytoplasmic tail, is induced on activated T and B lymphocytes (9, 13, 14). Its ligands, PD-L1 (B7-H1) and PD-L2, are expressed on APCs, activated T cells, and crucially, on a wide variety of non-hematopoietic parenchymal cells, with their expression often amplified by inflammatory cytokines (15–17). The foundational role of this axis in peripheral tolerance was unequivocally demonstrated in PD-1-deficient mice. On the C57BL/6 background, aged PD-1 knockout mice spontaneously developed a lupus-like disease characterized by proliferative arthritis, glomerulonephritis with IgG3 deposition, and splenomegaly (9, 14). On the BALB/c background, PD-1 deficiency led to a fatal autoimmune dilated cardiomyopathy, driven by high-titer IgG autoantibodies targeting a cardiomyocyte surface protein (18). These phenotypes, which were dependent on the adaptive immune system and markedly accelerated by concurrent Fas deficiency, positioned PD-1 as a non-redundant negative regulator essential for preventing systemic autoimmunity (9, 18). The foundational role of the PD-1 axis as a non-redundant ‘protective brake’ is unequivocally demonstrated by the spontaneous development of lupus-like glomerulonephritis in PD-1-deficient mice. This underscores that the presence and functional integrity of PD-1 signaling are essential requirements for maintaining peripheral tolerance and preventing the initial onset of systemic autoimmunity.

**Figure 1:**
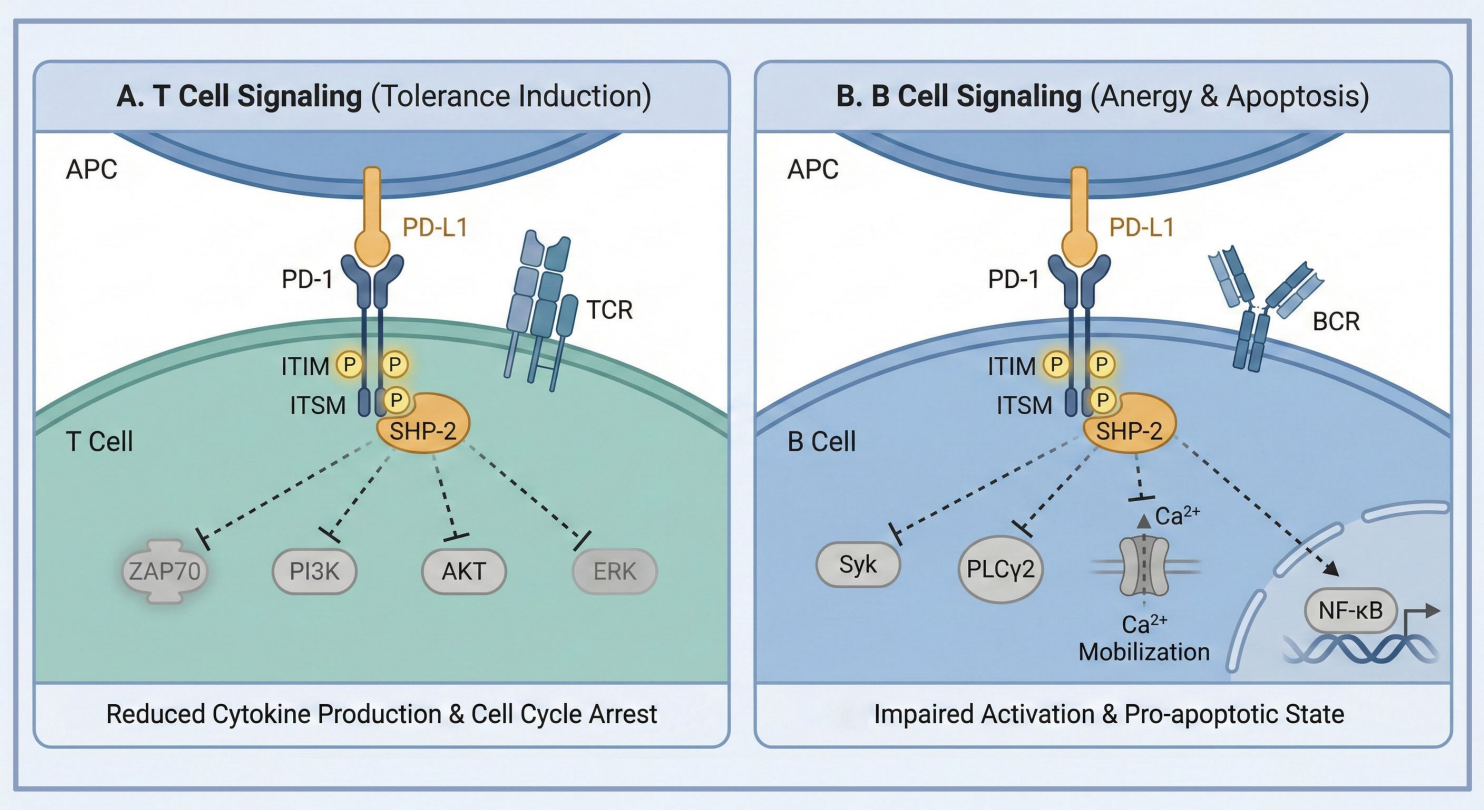
Intracellular inhibitory signaling pathways of the PD-1 axis in T and B cells. **(A)** T Cell Signaling: Upon ligation by PD-L1/PD-L2 and co-engagement with the TCR complex, the phosphorylated ITIM and ITSM motifs of PD-1 recruit the phosphatase SHP-2. This leads to the dephosphorylation of proximal signal transducers, including ZAP70 and the downstream PI3K/AKT/ERK pathways. **(B)** B Cell Signaling: Similarly, co-ligation with the BCR triggers SHP-2 recruitment, which dampens calcium (Ca^2+^) mobilization and inhibits effector molecules such as Syk and PLCγ2. Clarification: This scheme is a simplified representation of intracellular inhibitory events; in the physiological immunological synapse, these processes are integrated with TCR/BCR signaling and modulated by bidirectional interactions, including the cis-interaction between PD-L1 and CD80 (B7-1). APC, antigen-presenting cell; ITIM, immunoreceptor tyrosine-based inhibitory motif; ITSM, immunoreceptor tyrosine-based switch motif. Created with BioRender.com.

At the molecular level, PD-1 engagement delivers potent inhibitory signals that quell lymphocyte activation. Upon ligation by PD-L1 or PD-L2 and co-engagement with the antigen receptor, the phosphorylated tyrosine within PD-1’s cytoplasmic domain recruits the Src homology 2-domain-containing tyrosine phosphatase 2 (SHP-2) (16, 19). In T cells, this leads to the dephosphorylation of key signal transducers downstream of the T cell receptor (TCR), resulting in inhibited proliferation, reduced cytokine production, and cell cycle arrest (13, 16). This inhibitory function is context-dependent, being particularly potent at limiting responses to low-affinity or persistent antigen stimulation, a scenario akin to chronic infection or autoimmunity (13, 16). Importantly, PD-1 also exerts direct inhibitory control over B cells. Chimeric receptor studies demonstrated that the PD-1 cytoplasmic tail can recruit SHP-2 upon co-ligation with the B cell receptor (BCR), leading to dampened calcium mobilization and phosphorylation of effector molecules like Syk and PLCγ2 (19). Correspondingly, B cells from PD-1-deficient mice exhibit augmented proliferative responses to anti-IgM stimulation, elevated serum levels of specific IgG subclasses (notably IgG3), and enhanced antibody responses to T-independent antigens, highlighting PD-1’s role as a negative regulator of B cell proliferation, differentiation, and class-switch recombination (14) (**Figure 1**).

The PD-1 pathway orchestrates immunological tolerance through spatially and temporally distinct mechanisms. Its functions can be broadly categorized into the *induction* and the *maintenance* of peripheral tolerance (20). The induction phase primarily involves interactions in lymphoid organs. Here, PD-L1/PD-L2 expressed on professional APCs can deliver inhibitory signals to autoreactive T cells that have escaped central tolerance, thereby switching them off or promoting anergy (20, 21). Furthermore, PD-1 engagement is instrumental in the development and function of regulatory T cells (Tregs), a cornerstone of peripheral tolerance. The PD-1:PD-L1 interaction can promote the conversion of naive T cells into Foxp3^+^ Tregs, particularly in microenvironments rich in transforming growth factor-beta (TGF-β) (21, 22). Once generated, Tregs themselves require a network of inhibitory receptors for their full suppressive capacity; for instance, CTLA-4 is critical for Treg-mediated control of immune responses, illustrating the integrated nature of checkpoint control in sustaining tolerance (23, 24). PD-1 signaling thus helps to establish a regulatory milieu that raises the threshold for T cell activation, safeguarding against autoimmunity (21).

The maintenance of tolerance, or “tissue tolerance,” is largely mediated by PD-L1 expression on non-hematopoietic cells within peripheral tissues (17, 20). By engaging PD-1 on effector T cells that have infiltrated an organ, parenchymal PD-L1 directly suppresses local T cell function, thereby limiting immune-mediated tissue damage. This protective role is illustrated in the non-obese diabetic (NOD) mouse model of type 1 diabetes, where PD-L1 is expressed on inflamed pancreatic islets. Blockade of the PD-1:PD-L1 interaction rapidly precipitates diabetes by unleashing islet-reactive T cells, underscoring the tissue-protective function of this specific ligand-receptor pair in an autoimmune target organ (25). This model underscores a key principle: the expression pattern of PD-1 ligands dictates the fate of self-reactive T cells. PD-L1, with its broad inducible expression, appears particularly critical for controlling effector T cells in peripheral tissues and maintaining local tolerance (25, 26).

The PD-1 pathway does not operate in isolation but is part of a broader symphony of co-inhibitory and co-stimulatory signals that fine-tune immune responses (27). Its function can be influenced by other regulatory pathways. For instance, a subset of LAG3^+^ Tregs that suppresses humoral autoimmunity requires PD-1 expression on B cells to mediate suppression via TGF-β3, highlighting a cross-talk between the PD-1 pathway and other regulatory circuits in controlling B cell responses (28). Furthermore, the discovery of an interaction between PD-L1 and B7-1 (CD80) revealed an additional layer of complexity, demonstrating a bidirectional inhibitory signal that can modulate T cell activation beyond the classic PD-1:PD-L1 axis (17). This intricate network ensures robust tolerance but also creates multiple potential nodes for dysregulation. The genetic disruption of PD-1 in mice reveals a system where the loss of a single checkpoint is sufficient to unmask latent autoreactivity, leading to a T cell-dependent breakdown of B cell tolerance and the production of pathogenic autoantibodies, as seen in the lupus-like and cardiomyopathy models (5, 9, 18). This foundational biology—where PD-1 serves as a critical brake on both T and B cell responses to preserve self-tolerance—forms the essential backdrop against which its complex behavior in the established autoimmune environment of SLE must be understood.

## 3 Dysregulation of T cell subsets in SLE: Tfh, Tfr, and Tph modulated by PD-1/PD-L1

The generation of T follicular helper (Tfh) cells, defined by expression of the transcription factor Bcl-6, chemokine receptor CXCR5, and surface markers including inducible T-cell costimulator (ICOS) and PD-1, is essential for providing cognate help to B cells in germinal centers (GCs) (29–32). In SLE, this specialized help is co-opted to support autoreactive B cells. Multiple studies report an expansion of circulating Tfh-like cells (identified by phenotypes such as CXCR5^+^ICOS^+^PD-1^+^ or CXCR5^hi^PD-1^hi^) in patients, with their frequency correlating positively with disease activity, levels of anti-dsDNA antibodies, and circulating plasmablasts (33–35). This expansion is functionally significant, as these cells produce copious IL-21, a cytokine pivotal for B cell differentiation and pathogenic in SLE (36–38). The Tfh compartment itself is heterogeneous, comprising subsets such as Tfh1, Tfh2, and Tfh17 cells (39). Notably, a Tfh2 subset, characterized by GATA-3 and IL-4 expression, is spontaneously expanded in mice with T cell-specific deletion of Ets1 (a lupus-associated gene) and promotes IgE class switching; similar Tfh2 expansion correlates with disease parameters in human SLE (40). The development and positioning of Tfh cells are regulated by intricate signals. ICOS signaling is crucial not only for Tfh function but also for their initial follicular recruitment, a process shown to depend on ICOS ligand expressed by bystander B cells within the follicle (41). Consequently, therapeutic blockade of the ICOS pathway (B7RP-1) ameliorates autoimmunity in lupus-prone mice by reducing Tfh and GC B cell numbers (42). The PD-1/PD-L1 axis further fine-tunes this process. Dendritic cell (DC)-expressed PD-L1 is essential for limiting Tfh cell differentiation and their capacity to help B cells with class switching, illustrating a cell-type-specific regulatory function of this ligand (43). However, the high PD-1 expression characteristic of Tfh cells in SLE appears to mark a state of activation rather than exhaustion, contributing to the pathway’s paradoxical role.

Counterbalancing the help provided by Tfh cells are T follicular regulatory (Tfr) cells. Tfr cells represent a subset of Tregs that co-express Foxp3 and Bcl-6, enabling their migration to GCs via CXCR5 where they suppress Tfh cells and GC B cell responses (44–46). They are critical for preventing excessive or autoreactive antibody production. In SLE, this regulatory brake is defective. Patients exhibit decreased frequencies of circulating Tfr cells and an increased Tfh to Tfr ratio, which correlates more strongly with disease activity than Tfh cell frequency alone (47). The numerical deficiency is accompanied by functional impairment. Circulating Tfr cells in healthy individuals and those with ongoing humoral activity may indicate immune responses but often lack full B cell-suppressive capacity (48). In SLE, the defect is more pronounced. The impaired generation and function of Tfr cells are linked to IL-2 deficiency, a hallmark of SLE T cell biology (49, 50). IL-2 is critical for the maintenance and function of Treg cells, and low-dose IL-2 therapy in SLE patients can restore the Tfr/Tfh balance by promoting the conversion of Tfh cells into functional Tfr cells, a process involving STAT3/STAT5-mediated epigenetic regulation of Foxp3 and Bcl-6 loci (50, 51). Tfr cells exert suppression through multiple mechanisms, including the production of neuritin, a protein taken up by B cells that dampens plasma cell differentiation and IgE class switching (52). The PD-1 pathway also intersects with Tfr biology. While PD-1 signaling can promote the survival and suppressive function of conventional CD4^+^ Tregs (53), its specific role on Tfr cells within the GC context, potentially in concert with other checkpoints like CTLA-4, adds another layer of complexity to the control of humoral tolerance (54).

**Figure 2:**
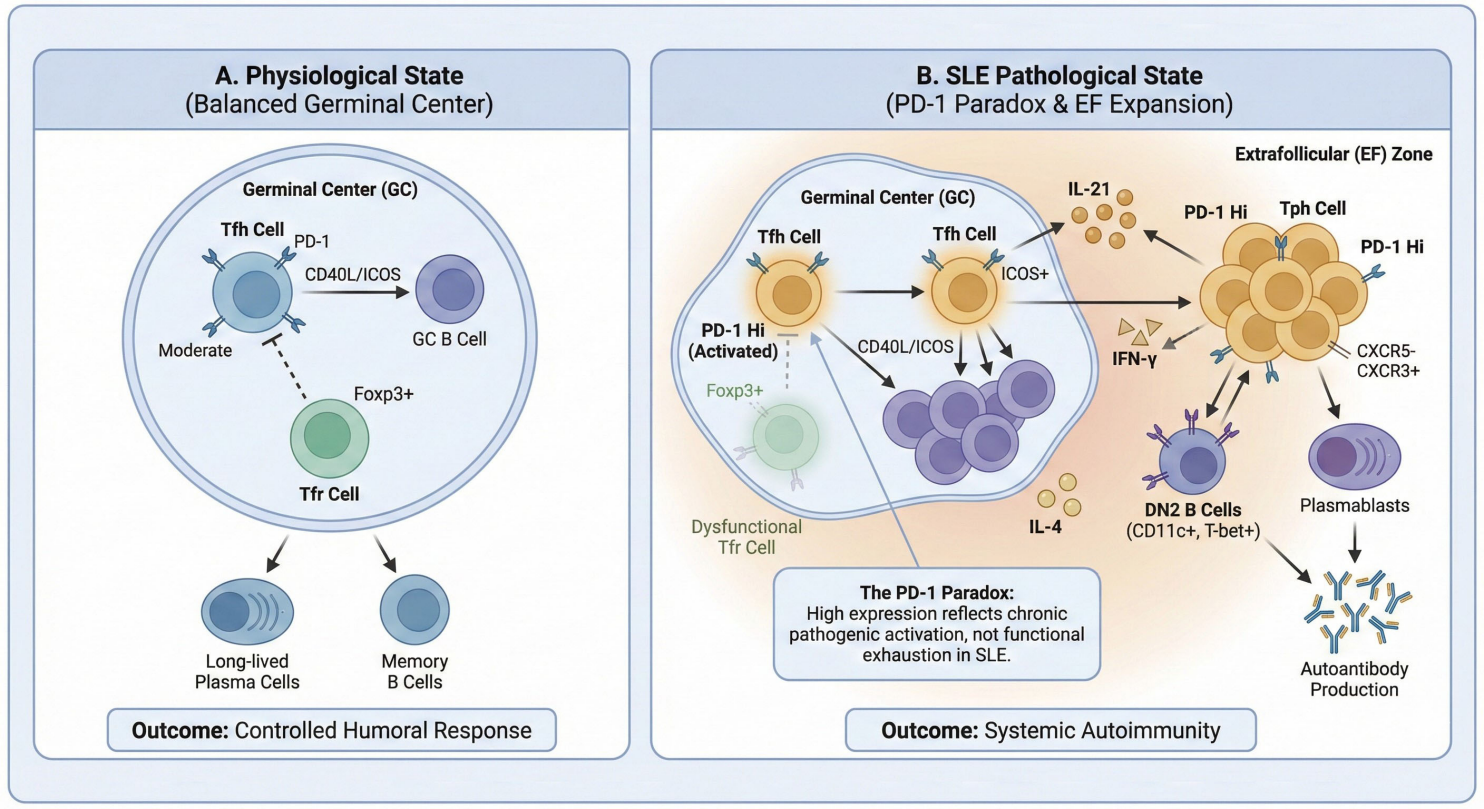
Dynamics of T-B cell interactions: physiological vs. SLE pathological states. **(A)** Physiological State: In a balanced GC, Tfh cells provide regulated help to B cells, while Tfr cells maintain follicular tolerance. **(B)** SLE Pathological State: This panel depicts the “PD-1 Paradox” where high PD-1 expression on pathogenic Tfh and Tph cells marks chronic activation rather than functional exhaustion. This dysregulation fuels the expansion of EF zones and the differentiation of DN2 B cells into autoantibody-secreting plasmablasts. DN2, double-negative 2 B cells; EF, extrafollicular; GC, germinal center. Created with BioRender.com.

The paradigm of T cell help has been further expanded by the identification of a distinct CXCR5-negative helper subset: T peripheral helper (Tph) cells. Defined as CD4^+^PD-1^hi^CXCR5^-^ T cells, Tph cells share the ability to produce B cell-helpful cytokines like IL-21 but provide help outside of follicles, potentially driving extrafollicular B cell responses which are prominent in SLE flares (6, 55). This population is markedly expanded in the circulation of SLE patients and its frequency strongly correlates with clinical disease activity, levels of autoantibodies, and the presence of CD11c^+^ age-associated B cells (55, 56). Notably, the correlation with disease activity can be stronger for Tph cells than for classical circulating Tfh cells (55). Tph cells are not merely bystanders; they are functionally potent, promoting plasmablast differentiation in a manner dependent on the transcription factor MAF and IL-21 (55). Their presence within inflamed tissues, such as lupus nephritis (LN) kidneys, where they correlate with local B cell infiltration, underscores their potential pathogenic role (55). The high expression of PD-1 on Tph cells epitomizes the paradox of this pathway in SLE: rather than signifying functional exhaustion, PD-1^hi^ marks a highly activated, tissue-invasive helper subset capable of fueling autoreactive B cell responses. Beyond marking a state of chronic activation, the elevated expression of PD-1 on Tfh and Tph cells in SLE may represent a compensatory but insufficient regulatory response to the potent pro-inflammatory signals generated within the autoimmune environment. Under the persistent stimulation of IFN and other inflammatory cytokines, the upregulation of PD-1 likely reflects the immune system’s attempt to re-establish a negative feedback loop. However, this ‘compensatory brake’ appears to be functionally subverted or simply overwhelmed by the magnitude of the inflammatory drive, a phenomenon that mirrors the inadequate expansion of regulatory Tfr cells seen during disease flares. Consequently, this failure of the PD-1 pathway to achieve functional suppression, despite high receptor levels, remains a central driver of the sustained production of pathogenic autoantibodies in SLE. This suggests that the functional outcome of PD-1 engagement is context-dependent, shifting from inhibition in tolerance to a marker of aberrant activation in chronic autoimmunity (**Figure 2**).

To resolve the “PD-1 Paradox” in SLE, it is imperative to distinguish it from the classical paradigm of immune exhaustion. Classical exhaustion is defined not merely by PD-1 upregulation, but by the sustained co-expression of multiple inhibitory receptors—such as CTLA-4, LAG-3, and TIM-3—coupled with a progressive loss of effector functions, including the reduced production of IL-2 and IFN-γ (57). However, in the SLE milieu, PD-1^hi^ Tfh and Tph cells do not exhibit functional quiescence. Instead, they remain highly potent, producing robust levels of IL-21 and IFN-γ to drive GC reactions and extrafollicular B cell expansion (58). This evidence suggests that PD-1^hi^ expression in SLE marks a state of chronic, pathogenic activation rather than terminal exhaustion (59). Furthermore, the inhibitory profile of these pathogenic cells is often incomplete or molecularly dysregulated. For instance, while circulating Tfr cells in SLE patients express high levels of PD-1, they simultaneously exhibit a significant downregulation of FoxP3 and CTLA-4. This specific molecular signature leads to impaired suppressive capacity rather than functional anergy. This “pseudo-exhausted” but functionally active phenotype is further substantiated by the presence of anti-PD-1 IgG autoantibodies in patients (60). These antibodies functionally block the PD-1 inhibitory signal to enhance T cell proliferation, demonstrating that the signaling axis remains a primary, though subverted, regulatory node. Finally, the clinical manifestation of lupus-like syndromes following immune checkpoint inhibitor (ICI) therapy confirms that the PD-1 axis continues to serve as a critical, albeit overwhelmed, guardian of peripheral tolerance even amidst established systemic inflammation (10, 61, 62).

The broader landscape of Treg cells in SLE also exhibits profound abnormalities that extend beyond the follicular niche. Numeric and functional deficits in CD4^+^CD25^+^Foxp3^+^ Tregs have been consistently reported in patients and murine models (63–67). These cells display increased fragility and impaired suppressive capacity (65, 68). The PD-1/PD-L1 axis is intimately involved in Treg homeostasis. In lupus-prone mice, PD-1 signaling on CD4^+^ Tregs is required for their resistance to apoptosis and maintenance of suppressive function; blockade of PD-1 consistently enhances the suppressive capacity of the remaining PD-1lo Tregs by releasing the inhibitory checkpoint on their regulatory function by altering their survival and functional profile (53). Furthermore, regulatory CD8^+^ T cells induced by tolerogenic regimens in lupus models upregulate Foxp3 while downregulating surface PD-1, and their suppressive function is dependent on this altered PD-1/Foxp3 dynamic (69). Other Treg subsets, such as IL-10-producing Tr1 cells identified by co-expression of CD49b and LAG-3, also contribute to the control of autoimmunity, and their interaction with the PD-1 pathway warrants further exploration (70). The imbalance extends to effector subsets, with an increased Th17/Treg ratio observed in active disease (71). This pan-T cell dysregulation, encompassing defective regulation and hyperactive help, creates a permissive environment for autoreactive B cells. Therapeutic strategies aiming to re-establish balance, such as low-dose IL-2 or nanoparticle delivery of IL-2/TGF-β to expand both CD4^+^ and CD8^+^ Tregs, show promise in preclinical models by directly targeting these T cell defects (51, 72).

In summary, the pathogenesis of SLE features a profound dysregulation of T cell subsets that guide humoral immunity. An expansion of hyper-functional Tfh and Tph cells, coupled with a numerical and functional deficiency in Tfr and other Treg populations, disrupts the checks and balances required for maintaining B cell tolerance. The PD-1/PD-L1 axis is deeply embedded in this dysregulation, modulating the differentiation, migration, and functional output of these critical T cell subsets. Its role transitions from a guardian of tolerance in homeostasis to a complex participant in disease, where high PD-1 expression can paradoxically mark pathogenic helper T cells while its signaling remains crucial for the stability of the impaired regulatory compartment. This intricate interplay positions the PD-1 pathway and its cellular effectors as central determinants of the aberrant B cell responses that define SLE.

## 4 The dual role of PD-1/PD-L1 in B cell biology: from exhaustion to aberrant activation in SLE

The dysregulated T cell landscape, intricately modulated by PD-1 signaling, directly shapes the fate and function of B cells in SLE. While PD-1/PD-L1 interactions are classically understood as inhibitory checkpoints, their role in B cell biology reveals a profound and context-dependent duality. In physiological settings, this axis is a critical governor of B cell activation and terminal differentiation, serving to constrain excessive humoral responses. In the autoimmune milieu of SLE, however, this regulatory circuitry is subverted, contributing to both the functional impairment of regulatory subsets and the paradoxical hyper-activation of pathogenic effector B cell populations.

At a fundamental level, PD-1 delivers potent negative signals directly within B cells. Upon co-engagement with the BCR, the phosphorylated cytoplasmic tail of PD-1 recruits the phosphatase SHP-2, leading to the dephosphorylation of key downstream signaling molecules such as Syk, PLCγ2, and ERK, thereby dampening BCR-induced activation, calcium mobilization, and proliferation (19). This inhibitory function is evident in PD-1-deficient mice, which exhibit splenomegaly, augmented B cell proliferative responses to anti-IgM, and elevated serum levels of particular IgG subclasses, especially IgG3, in response to T cell-independent type 2 antigens (14). The regulation extends to innate-like B-1 cells, where PD-1 suppresses their expansion and long-lived IgG production following antigen encounter (73), and where its ligand PD-L2 modulates natural antibody production through an IL-5-dependent mechanism (74). Within GCs, PD-1 ligands expressed on B cells engage PD-1 on Tfh cells, an interaction crucial for sustaining Tfh cell cytokine production and ensuring the survival of high-affinity, long-lived plasma cells (75). Thus, under homeostatic conditions, the PD-1 pathway fine-tunes B cell responses across multiple subsets and anatomical compartments, preventing excessive or inappropriate antibody output.

In SLE, this carefully balanced system is fundamentally rewired. B cells from patients exhibit altered baseline expression and inducible capacity of PD-1 and its ligands. A consistent finding is the increased expression of PD-1 on unstimulated lupus B cells (76). More importantly, upon activation via combinations of stimuli such as CD40L, IL-2/IL-10, CpG, and BCR engagement, SLE B cells demonstrate a significantly diminished capacity to upregulate PD-L1 compared to healthy B cells (76). This defective PD-L1 upregulation is inversely correlated with the interferon signature and clinical disease activity, suggesting that an impaired ability to engage the PD-1 inhibitory pathway on interacting T cells may contribute to unchecked T cell help. Concurrently, the expression of PD-L1 on CD19^+^ B cells is elevated in SLE patients and positively correlates with disease activity indices (77). *In vitro*, the amounts of anti-dsDNA and immunoglobulin G secreted by CD19^+^PD-L1^+^ B cells from SLE patients differ from those secreted by their healthy counterparts, and this population correlates strongly with circulating Tfh cells (77). This phenotype of high PD-1 with functionally diminished PD-L1 responsiveness creates a scenario where lupus B cells may be less susceptible to intrinsic PD-1-mediated inhibition while simultaneously using PD-L1 to modulate the local T cell microenvironment.

The duality of PD-1/PD-L1 is particularly evident in the context of specific pathogenic B cell subsets that expand in SLE. The disease is characterized by a prominent extrafollicular response, giving rise to effector B cells that often exhibit an atypical or exhausted phenotype. One such subset is the double-negative (DN2, IgD^-^CD27^-^CD11c^+^) B cell, which represents a pre-plasma cell population enriched in patients with active disease and nephritis (12). These cells, along with related age-associated B cells defined by T-bet and CD11c expression, are key drivers of autoimmunity (78, 79). They emerge from activated naive B cells and are characterized by hyper-responsiveness to innate signals like TLR7, a transcriptomic network involving T-bet and AP-1, and a metabolic state marked by hyperactivation of mTORC1 (12, 80, 81). While exhibiting markers of activation and differentiation, these cells also display functional dysregulation, impaired antigen presentation, and accelerated apoptosis (81). The PD-1 axis is interwoven with their biology; for instance, the generation of T-bet^+^ age-associated B cells is regulated by pathways involving IRF5 and IL-21, cytokines that are themselves implicated in PD-1/PD-L1 dynamics (82). Furthermore, the extrafollicular differentiation of autoreactive B cells into antibody-secreting cells is heavily dependent on help from Tph cells and cytokines like IL-21, pathways where PD-1/ICOS interactions play a coordinating role (83–85). Thus, PD-1 signaling may be involved in both the initial dysregulated activation of these precursors and in subsequent attempts by the immune system to control these aberrant effector populations.

This paradox extends to the regulatory B cells (Breg) compartment. In healthy individuals, CD24^hi^CD38^hi^ immature B cells possess regulatory capacity, suppressing T helper 1 cell differentiation via IL-10 in a process promoted by pDCs through IFN-α and CD40 engagement (86, 87). This pDC-Breg cross-talk forms a feedback loop, as Bregs in turn restrain pDC IFN-α production via IL-10. In SLE, this regulatory circuit is shattered. pDCs from patients fail to induce functional IL-10-producing Bregs from CD24^hi^CD38^hi^ B cells, instead skewing their differentiation toward plasmablasts (87). The defective Breg induction is associated with altered STAT1/STAT3 signaling and can be recapitulated by exposing healthy B cells to high doses of IFN-α, a hallmark of SLE. While the direct role of PD-1/PD-L1 in this specific defect is less defined, the broader disruption of co-inhibitory checkpoint balance is evident. The impaired function of Bregs, a subset known to interact with and regulate Tfh and Tfr cells (88), removes a vital layer of control over GC and extrafollicular reactions.

Therapeutic interventions further illuminate the complex role of the PD-1 axis in SLE B cell pathophysiology. B cell depletion with anti-CD20 (e.g., rituximab) remains a cornerstone therapy, demonstrating that removing CD20^+^ B cell precursors, including autoreactive naive and memory subsets, can improve disease activity (89). Interestingly, rituximab may also modulate remaining B cells, enriching for an activated naïve phenotype and altering their T cell stimulatory capacity toward a more Th2-like profile, effects that could involve changes in co-stimulatory and co-inhibitory ligand expression (90). The success of broader B lineage targeting with anti-CD19 agents (e.g., inebilizumab), which also deplete plasmablasts and some plasma cells, underscores the contribution of later differentiation stages (91, 92). Strategies to directly manipulate the PD-1 pathway are being informed by these complexities. For example, human mesenchymal stem cells (hMSCs) can ameliorate lupus in models, and their suppressive effect on B cells is enhanced by priming with agents like phorbol esters. This enhanced suppression is mediated, in part, through upregulated PD-L1 on hMSCs engaging PD-1 on B cells to induce apoptosis (93). Similarly, murine BM-MSCs inhibit lupus B cell activation in an IFN-γ and PD-1/PD-L1-dependent manner (94). Conversely, the natural breakdown of tolerance in SLE can itself generate autoantibodies against checkpoint molecules. Elevated levels of IgG autoantibodies against PD-1 have been found in new-onset SLE patients, correlating with disease activity. These anti-PD-1 autoantibodies can functionally enhance T cell proliferation *in vitro*, effectively blocking the co-inhibitory signal and thereby exacerbating immune activation (95). This finding provides a stark illustration of the critical regulatory importance of the PD-1/PD-L1 axis: while therapeutic augmentation of this signaling pathway can restore immune restraint, its functional disruption by the disease process itself (e.g., via autoantibodies) removes this essential brake, thereby driving pathology. Ultimately, the PD-1/PD-L1 axis in SLE B cell biology acts as a consistent inhibitory interface, whose clinical outcome depends on the cellular context and whether the checkpoint’s functional integrity is maintained or compromised. It functions as a dynamic interface whose outcome depends on the cellular subset involved, the differentiation state, the tissue microenvironment, and the concomitant signals from cytokines and other receptors. In SLE, genetic and epigenetic programming establishes a B cell compartment poised for extrafollicular effector responses (80), within which dysregulated PD-1/PD-L1 expression and function contribute to a loss of intrinsic B cell restraint, a failure of regulatory feedback, and the sustenance of pathogenic T-B interactions. Resolving this paradox—distinguishing when PD-1 signaling represents a failed attempt at exhaustion versus an active contributor to aberrant activation—is critical for designing precise immunotherapies that can recalibrate this checkpoint to restore tolerance without causing broad immunosuppression.

## 5 Contributions of PD-L1^+^ innate immune cells and stromal cells to SLE pathogenesis

The dysregulated crosstalk between T and B lymphocytes, modulated by the PD-1/PD-L1 axis, forms a core pathogenic circuit in SLE. However, the pathogenesis extends beyond this adaptive immune dyad, involving a complex network of innate immune cells and stromal elements that express PD-L1 and other immunomodulatory molecules. These cells are not merely bystanders; they actively shape the inflammatory microenvironment, influence adaptive immune responses, and contribute directly to tissue damage. Their functional states, often abnormal in SLE, can be both a cause and a consequence of the breakdown in tolerance, creating feedback loops that sustain disease.

Neutrophils emerge as pivotal players with multifaceted roles. In SLE patients, a distinct subset of neutrophils expressing PD-L1 is significantly expanded, and its frequency correlates positively with disease activity and severity markers such as SLE Disease Activity Index (SLEDAI) score and inflammatory cytokines (96). These PD-L1^+^ neutrophils possess immunomodulatory capacity. Inflammatory stimuli like IFN-γ can induce PD-L1 expression on neutrophils, enabling them to suppress lymphocyte proliferation in a PD-L1-dependent manner (97). This suggests a potential feedback mechanism where neutrophils may attempt to constrain excessive T cell activation. Conversely, neutrophils that migrate to draining lymph nodes can enhance CD4^+^ T cell responses while simultaneously curbing their expansion via a PD-L1-dependent checkpoint, indicating a dual role in regulating adaptive immunity (98). Beyond checkpoint modulation, neutrophils contribute to pathogenesis through alternative pathways. The presence of low-density granulocytes (LDGs) in SLE is associated with a proinflammatory phenotype, enhanced capacity to synthesize type I IFNs and TNF-α, and direct cytotoxicity toward endothelial cells, linking them to vascular damage and impaired repair (99). Furthermore, neutrophil extracellular traps (NETs) provide a source of autoantigens and can incite inflammation, with impaired clearance of such apoptotic and necrotic material being a fundamental defect in SLE that perpetuates autoimmunity (100–102). The chemokine axis, particularly CXCR4 and its ligand SDF-1, is crucial for neutrophil egress from and return to the bone marrow; dysregulation here may affect circulating neutrophil homeostasis and their recruitment to tissues like the kidney (103, 104).

Basophils, though rare, have been implicated in SLE pathogenesis through mechanisms intimately linked to the PD-1/PD-L1 axis and T cell help. In both human SLE and murine models, basophils accumulate in secondary lymphoid organs (105, 106). They become activated and upregulate PD-L1 (105–107). This PD-L1 expression on basophils is functionally critical. PD-L1^+^ basophils, along with their production of IL-4, are directly responsible for the pathogenic accumulation, maintenance, and function of autoreactive Tfh and Tfh2 cells in lymph nodes, thereby promoting autoantibody production and LN (106). This establishes a direct mechanistic link wherein basophils, via PD-L1 and IL-4, license pathogenic Tfh cells. The activation of basophils is correlated with disease activity and nephritis in humans (105, 108). Their reduction in the peripheral blood of SLE patients may reflect this active recruitment to disease sites (109). The cytokine milieu, particularly IFN-γ in the context of IL-3 priming, can further induce PD-L1 on human basophils, potentially integrating them into the broader IFN-rich environment of SLE (107). The stark contrast between PD-L1^+^ neutrophils and basophils provides a definitive example of the PD-1/PD-L1 axis’s dual nature in SLE. While neutrophils utilize PD-L1 to exert an anti-inflammatory, suppressive effect on T-cell proliferation, basophils exploit the same molecule to drive a pro-inflammatory program by promoting the maintenance of pathogenic Tfh cells and subsequent autoantibody production. This functional divergence underscores that the immunological outcome of the PD-1/PD-L1 interaction is not fixed but is fundamentally rewired by the cellular and microenvironmental context within the SLE milieu.

Other innate immune populations also contribute. Monocytes and macrophages, upon engagement of PD-1 by PD-L1, can produce IL-10, leading to reversible CD4^+^ T cell dysfunction—a mechanism described in chronic viral infection that may have parallels in SLE’s hyperimmune activation (110). pDCs, major producers of IFN-α, are central to SLE pathogenesis. Their development can be potently inhibited by MSCs in a cell contact-dependent manner, a regulatory interaction that may be disrupted in disease (111). Furthermore, unconventional T cells like Vδ2 T cells show altered distribution and an activated, proinflammatory phenotype in SLE, with increased infiltration into kidneys and heightened expression of cytokines and homing receptors (112).

**Figure 3:**
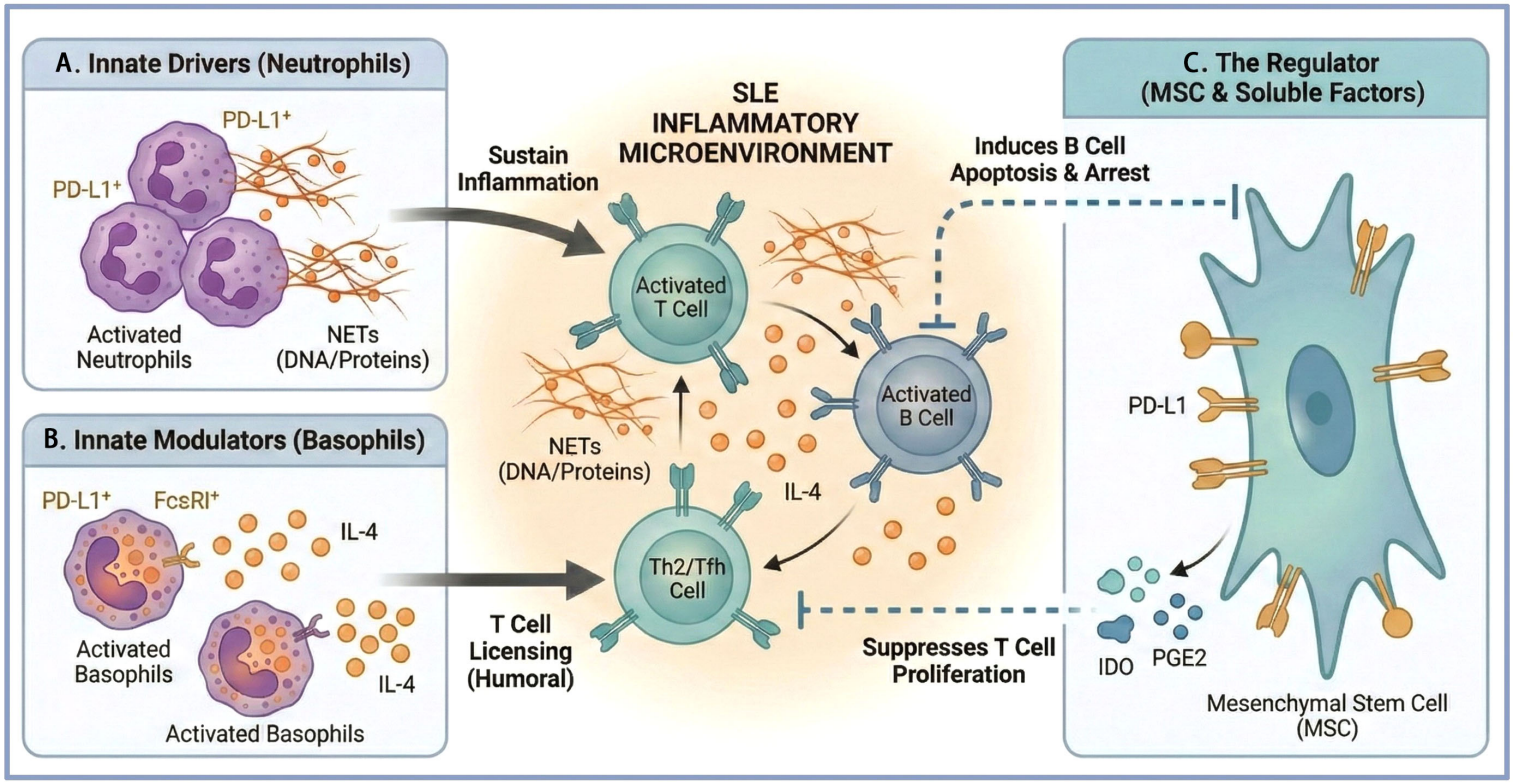
Cellular drivers of the SLE inflammatory microenvironment and immunomodulation by MSCs. **(A)** Innate Drivers: Activated PD-L1^+^ neutrophils contribute to sustained inflammation through NETs. **(B)** Innate Modulators: PD-L1^+^ basophils “license” pathogenic humoral responses by promoting Th2/Tfh cell accumulation. **(C)** The Regulator: MSCs exert potent immunomodulatory effects by inducing B cell apoptosis and suppressing T cell proliferation through PD-L1-dependent contact and soluble factors like IDO and PGE2. Created with BioRender.com. IDO, indoleamine 2,3-dioxygenase; MSC, mesenchymal stem cell; NETs, neutrophil extracellular traps; PGE2, prostaglandin E2. Created with BioRender.com.

Stromal cells, particularly MSCs, represent a potent regulatory node within the bone marrow and peripheral tissues. MSCs possess broad immunomodulatory abilities, suppressing T cell proliferation and inducing T cell apoptosis via mechanisms involving FAS-L and PD-L1 (93, 113). Their impact on B cells is context-dependent but significant. hMSCs can inhibit B cell proliferation, differentiation into antibody-secreting cells, and chemotactic responses through soluble factors (114). In the setting of SLE, murine MSCs inhibit BCR-dependent activation of both follicular and marginal zone B cells; this suppression is dependent on IFN-γ and cell contact, involving the PD-1/PD-L1 pathway (94) (**Figure 3**). Priming or “licensing” of MSCs by inflammatory signals enhances their immunosuppressive capacity. IFN-γ-preconditioned hMSCs gain the ability to inhibit B cell function and attract B cells via CXCL10 (115). Similarly, phorbol ester treatment renders hMSCs capable of inducing B cell apoptosis in a PD-L1-dependent manner (93). These mechanisms underscore the potential of MSCs as a therapy, an approach supported by meta-analyses showing efficacy in reducing proteinuria, autoantibodies, and renal pathology in animal models of LN and some clinical improvements in patients (116, 117). The therapeutic effect of MSCs can be synergistic with conventional immunosuppressants like prednisone or mycophenolate mofetil without compromising MSC function (118). Novel engineered MSC products, such as HXB-319, designed to enhance potency, show promise in improving survival and halting glomerulonephritis progression in murine lupus by modulating key immune subsets including PD-L1^+^ CD4^+^ T cells and Th17 cells (119).

The gut-kidney axis and environmental factors further illustrate the systemic nature of SLE pathogenesis where innate and stromal elements are involved. Dysbiosis of gut microbiota, marked by depletion of Lactobacillales, promotes renal disease in lupus-prone mice; restoration of a healthy microbiota can ameliorate nephritis by reducing inflammatory cytokines and skewing the Treg-Th17 balance (120). Supplementation with *Lactobacillus acidophilus* can synergize with tacrolimus therapy, partly by inducing immunomodulatory molecules like PD-L1 and IL-10 in a SIGNR3-dependent pathway (121). Even ambient exposure to certain environmental chemicals like quaternary ammonium compound disinfectants can alter immune phenotypes, reducing neutrophil migration and increasing PD-L1 expression on splenic neutrophils, which in turn modulates T cell activation and apoptosis, unexpectedly ameliorating splenomegaly in a lupus model (122).

In summary, the pathogenesis of SLE is orchestrated by a concert of cells beyond T and B lymphocytes. PD-L1-expressing neutrophils and basophils directly engage with adaptive immunity through checkpoint molecules and cytokines, driving Tfh responses and tissue injury. Stromal cells like MSCs serve as critical regulators, but their function may be subverted or insufficient in the disease state. The recruitment, activation, and functional programming of these innate immune and stromal cells are influenced by the SLE milieu—characterized by type I and II interferons, immune complexes, and apoptotic debris—and in turn, they reinforce this pathogenic environment. Therapeutic strategies that recalibrate the functions of these cells, either by enhancing regulatory mechanisms or inhibiting their pathogenic contributions, offer promising avenues for intervention that complement direct targeting of the adaptive immune axis.

## 6 PD-1/PD-L1 axis correlates with disease activity: circulating cellular biomarkers and genetic associations

The pathogenic interplay between innate immune cells, stromal elements, and the adaptive immune system in the SLE milieu not only shapes cellular phenotypes but also imprints distinct molecular signatures that correlate with clinical disease. The expression patterns of PD-1 and PD-L1 on various circulating immune cell subsets, along with genetic variations in the PDCD1 locus, have emerged as quantifiable biomarkers reflecting disease activity, organ involvement, and therapeutic response, thereby offering a window into the dysregulated checkpoint landscape of SLE.

The genetic foundation of PD-1 pathway dysregulation in SLE is underscored by polymorphisms within the PDCD1 gene. A seminal study identified an intronic single nucleotide polymorphism (PD1.3, rs11568821) that alters a RUNX1 binding site and is associated with increased susceptibility to SLE in European and Mexican populations (123). This association exhibits population-specific effects, being significant in Latin American and some European cohorts but not in Spanish or African populations, highlighting the influence of haplotype structure (124, 125). Importantly, the PD1.3A allele is not merely a risk factor for SLE susceptibility per se but shows a stronger association with specific severe phenotypes, particularly renal involvement and LN (124, 126). Mechanistically, the PD1.3A allele correlates with lower expression levels of the PD-1 receptor on activated CD4^+^ T cells, especially on CD4^+^CD25^+^ populations (127). Another polymorphism, PD1.5C, is also associated with SLE susceptibility in Europeans (124). These genetic findings provide a heritable basis for the suboptimal PD-1-mediated regulation observed in patients.

In the peripheral blood, the imbalance between helper and regulatory CD4^+^ T cell subsets expressing PD-1 serves as a dynamic cellular biomarker. Circulating T follicular helper (cTfh) cells, identified by co-expression of CXCR5 and high levels of PD-1 and ICOS, are expanded in SLE patients (34, 128, 129). Their frequency and, more specifically, the intensity of PD-1 expression on these cells, correlate positively with the SLEDAI, levels of autoantibodies (anti-dsDNA), and circulating plasmablasts (34, 35, 129). The functional competence of these cells is further indicated by their expression of the master transcription factor Bcl-6, which itself correlates with SLEDAI and is inducible by IL-21 (35). The pathogenic help provided by these Tfh cells to B cells is critically dependent on CD40L signaling (128). Recent attention has focused on a CXCR5-negative counterpart, the Tph cell, defined as CD4^+^CD45RA^-^CXCR5^-^PD-1high. This subset is markedly expanded in SLE circulation, shows a strong positive correlation with SLEDAI scores and multiple organ manifestations (nephritis, arthritis, serositis), and is associated with plasma cell generation (56, 130). A specific CXCR3-PD-1^+^ CD4^+^ T cell subset, independent of CXCR5 expression, also correlates with SLEDAI and autoantibody levels, positioning it as a sensitive indicator of disease activity (131). Conversely, the regulatory counterpart, Tfr cells (CD4^+^CXCR5^+^FoxP3^+^), exhibits a more complex relationship. While some studies report a decreased Tfr frequency and an increased Tfh/Tfr ratio that correlates with disease activity (47), others find an expansion of circulating Tfr cells in active SLE, potentially as a compensatory yet insufficient response (132). Critically, PD-1^+^ Tfr cells from SLE patients display functional impairment in suppressing Tfh cells, linked to decreased FoxP3, CTLA-4, and IL-2Rα expression, and this dysfunction correlates with anti-dsDNA levels and disease activity (133). The ratio of Tfr to Tfh cells in blood may also inform about ectopic lymphoid structure formation, as shown in related autoimmune conditions (134).

B cells themselves exhibit altered PD-1/PD-L1 expression profiles linked to disease activity. The frequency of CD19^+^PD-L1^+^ B cells in peripheral blood is elevated in SLE patients and significantly associated with higher disease activity indices (77). These PD-L1^+^ B cells demonstrate a greater capacity to secrete IgG and anti-dsDNA antibodies *in vitro* and their frequency correlates with that of pathogenic Tfh cells (77). Furthermore, lupus B cells display a paradoxical activation phenotype characterized by heightened baseline PD-1 expression but a diminished capacity to upregulate PD-L1 upon stimulation *in vitro*; this defective PD-L1 upregulation inversely correlates with the interferon signature and SLEDAI score (76). This phenotype is associated with reduced B cell proliferation, suggesting a state of aberrant regulation and hyporesponsiveness. Another expanded B cell subset in active SLE, the atypical memory B cell (AtM), is characterized by mTORC1 hyperactivation and functional dysregulation, and these cells accumulate in the kidneys of LN patients (81).

The biomarker potential of the PD-1/PD-L1 axis extends beyond the adaptive immune system. PD-L1-expressing neutrophils are increased in SLE, and their frequency correlates with SLEDAI, autoantibody titers, and inflammatory markers, decreasing after effective immunosuppressive treatment (96). Similarly, a distinct population of TIM-3^+^ PD-1^+^ natural killer (NK) cells is expanded in SLE and correlates with disease activity and severity, including serological markers and organ involvement (135). The presence of autoreactive IgE antibodies, which correlate with disease activity and nephritis, implicates an additional layer of humoral dysregulation (136). Furthermore, autoantibodies targeting the co-inhibitory receptors themselves have been identified. Elevated serum levels of anti-PD-1 IgG autoantibodies are found in new-onset SLE patients, correlate with SLEDAI and multiple organ involvements, and functionally enhance T cell proliferation *in vitro*, thereby subverting a key negative regulatory signal (95). This mirrors findings of anti-B7-H1 (PD-L1) autoantibodies in rheumatoid arthritis (RA) that can costimulate T cells (137).

These circulating cellular and serological biomarkers find their tissue correlate in affected organs. In LN kidneys, single-cell analyses and immunohistochemistry reveal a complex infiltrate including PD-1 expressing T cell subsets and *in situ* lymphoid structures supporting B cell activation (103, 138, 139). The presence of these organized structures is associated with more severe tubulointerstitial inflammation. The functional link between circulating Tph cells and renal plasmablasts, and between renal-infiltrating and lymph node Tfh cells, underscores the systemic nature of this dysregulated help (56, 140).

Collectively, the quantification of PD-1 and PD-L1 on specific circulating immune cell subsets (Tfh, Tph, Tfr, B cells, neutrophils, NK cells), alongside serological autoantibodies against checkpoint molecules and predisposing genetic variants, provides a multi-parametric framework to assess SLE disease activity, predict organ involvement (particularly nephritis), and potentially monitor therapeutic efficacy. This correlation underscores the central role of a dysregulated PD-1/PD-L1 axis in sustaining the pathogenic immune responses that drive clinical disease.

## 7 The PD-1/PD-L1 axis across the immune spectrum: from general autoimmunity to the oncology paradox

The programmed cell death protein 1 (PD-1) pathway, comprising the PD-1 receptor and its ligands PD-L1 and PD-L2, is a cornerstone of peripheral immune tolerance. Its primary function is to deliver co-inhibitory signals that dampen T-cell activation, prevent autoimmunity, and maintain immune homeostasis (141–144). Under physiological conditions, the PD-1/PD-L1 interaction is indispensable for safeguarding the body against self-reactivity (143, 145). This pivotal role is underscored by the development of spontaneous autoimmune phenotypes in PD-1-deficient mice (142, 143).

The protective role of the PD-1 axis is a common thread across diverse autoimmune landscapes (146, 147). Genetic studies have linked single nucleotide polymorphisms (SNPs) in the PDCD1 gene to an increased risk of developing multiple conditions, including SLE, RA, and type 1 diabetes (22, 143, 148). Beyond lymphoid organs, the axis mediates “tissue tolerance,” a concept vividly illustrated in the NOD mouse model (149, 150). In this model, PD-L1 expression on pancreatic islet cells is essential for protecting the organ from autoimmune attack (150). Conversely, a profound deficiency in PD-L1 expression—as observed in giant cell arteritis (GCA)—creates a state of “lost inhibition”. This allows PD-1^+^ T cells to receive unopposed stimulatory signals, leading to runaway autoimmunity and vasculitis (151, 152). These findings underscore that a functional PD-1/PD-L1 checkpoint is a non-redundant shield required to prevent the breakdown of self-tolerance (142, 145).

The physiological role of PD-1 as an immune brake is often subverted in malignancy, creating a profound therapeutic paradox when compared to SLE (147). Many cancers upregulate PD-L1 to induce T-cell exhaustion and functional impairment (146, 147, 153). This forms the basis for checkpoint blockade, where monoclonal antibodies restore anti-tumor immunity by “releasing the brake” (154–156). In contrast, the “PD-1 paradox” in SLE represents a state of inadequate or dysfunctional checkpoint signaling (10, 157, 158). In the pro-inflammatory milieu of SLE, high PD-1 expression on pathogenic subsets like Tfh and Tph cells marks a state of persistent activation rather than exhaustion (159–161). Consequently, SLE may necessitate therapeutic “agonism” to reinstate immune regulation (162, 163).

**Figure 4:**
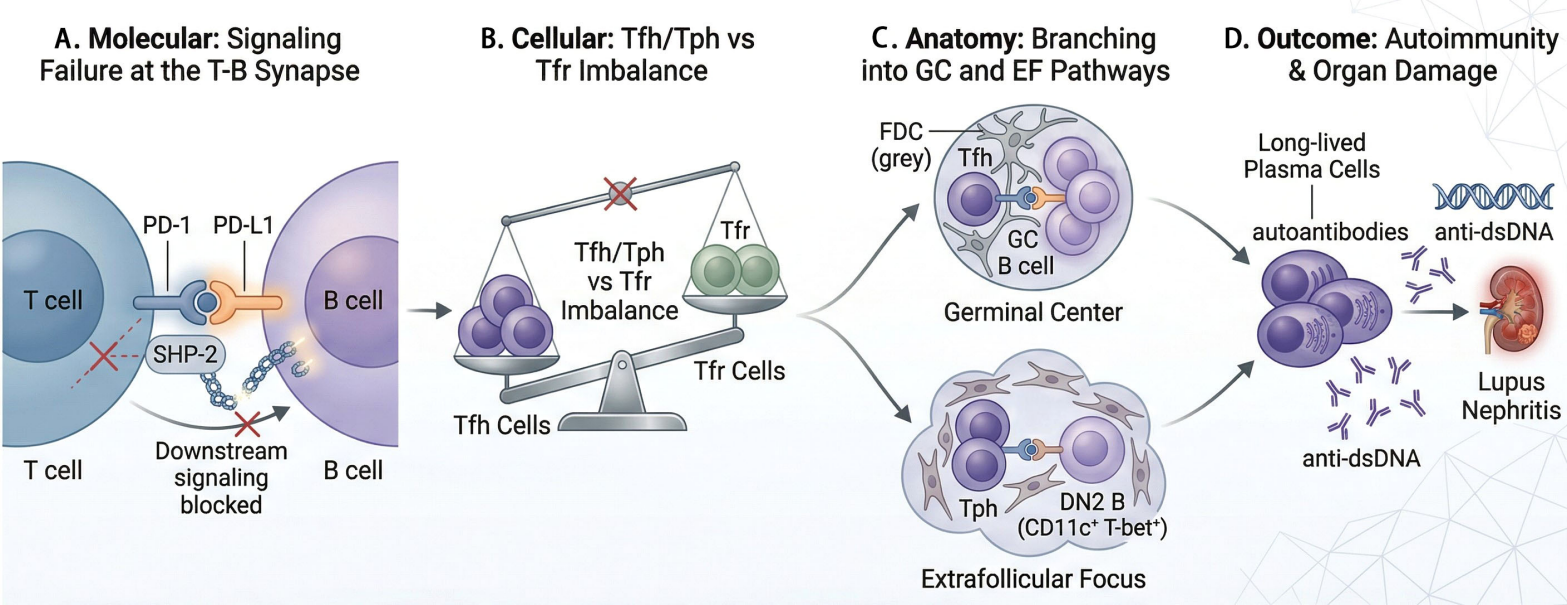
Conceptual framework of PD-1-mediated immune dysregulation and B-cell autoimmunity in SLE. **(A)** Molecular Level: In the SLE milieu, PD-1 signaling fails at the T-B synapse; the inability to recruit SHP-2 results in uninhibited downstream signaling, bypassing the immunological “brake”. **(B)** Cellular Level: This signaling defect precipitates an imbalance in the follicular niche, characterized by the expansion of pathogenic Tfh/Tph cells and the functional impairment of Tfr cells. **(C)** Anatomical Level: The dysregulated help branches into two distinct pathways: the GC pathway (driven by Tfh cells) and the EF focus (driven by Tph and DN2 B cells), both of which are hyper-activated in SLE. **(D)** Outcome: These synergistic pathways converge on the massive production of anti-dsDNA autoantibodies by plasmablasts, ultimately leading to systemic inflammation and organ-specific damage, such as Lupus Nephritis. DN2, double-negative 2 B cells; EF, extrafollicular; FDC, follicular dendritic cell; GC, germinal center. Created with BioRender.com.

This paradox finds its most direct clinical expression in the development of lupus erythematosus as an irAE following ICI therapy (164–166). Removing the PD-1-mediated brake can lower the threshold for immune activation against self-antigens, precipitating *de novo* SLE or subacute cutaneous lupus erythematosus (SCLE) (165–167). This iatrogenic phenomenon serves as a living model of the axis’s dual nature: its blockade during cancer therapy can trigger the very autoimmune condition linked to its functional insufficiency (165, 166). The dysregulation of PD-1-expressing T helper subsets thus represents the common mechanistic thread linking the intrinsic loss of tolerance in SLE to the iatrogenic breakdown induced by ICI therapy (158) (**Figure 4**).

## 8 Therapeutic targeting of the PD-1/PD-L1 axis and related pathways in SLE: from preclinical models to clinical trials

The strong correlation between a dysregulated PD-1/PD-L1 axis and SLE disease activity logically positions this pathway as a prime therapeutic target. However, translating this knowledge into effective treatments requires navigating the pathway’s complex, context-dependent biology, which necessitates a multi-pronged approach ranging from direct modulation of the checkpoint to combinatorial strategies and the induction of regulatory circuits.

Proof-of-concept for therapeutic manipulation in SLE was firmly established in preclinical models. In young, premorbid (NZB x NZW)F1 (BWF1) mice, *in vivo* administration of a neutralizing anti-PD-1 antibody paradoxically protected from LN and improved survival (168). This protection was associated with the maintenance and enhanced function of suppressive CD8^+^Foxp3^+^ T cells, which could suppress T helper cells and pathogenic B cells (168). Similarly, in BWF1 mice, systemic administration of a PD-L1-Ig fusion protein to engage and activate PD-1 on CD4^+^ T cells suppressed Th17 formation, reduced autoantibodies and cytokines, ameliorated renal injury, and prolonged survival (169). These studies highlight that the net effect of PD-1/PD-L1 manipulation depends on timing, cellular context, and the specific ligand-receptor interaction targeted, with both checkpoint blockade and engagement showing therapeutic potential in different experimental settings. Notably, the outcomes of PD-1/PD-L1 blockade exhibit striking discrepancies between murine models and human clinical practice. In young (NZB x NZW)F1 mice, neutralizing anti-PD-1 antibodies have paradoxically shown protective effects by enhancing the suppressive activity of CD8^+^Foxp3^+^ Tregs that restrain pathogenic B cell help. Conversely, in human oncology, systemic PD-1 blockade frequently triggers *de novo* SLE or exacerbates pre-existing autoimmunity as immune-related adverse events (irAEs). This cross-species divergence suggests that the functional outcome of PD-1 blockade is highly contingent upon whether the treatment releases the brake on effector populations or inadvertently promotes regulatory subsets within a specific immunological landscape. This concept is further supported by work with the tolerogenic peptide pConsensus (pCons). Tolerization of BWF1 mice with pCons induced CD8^+^ and CD4^+^ Tregs that suppressed autoimmunity, a process linked to increased intracellular Foxp3 while decreasing surface PD-1 expression on the induced CD8^+^ suppressors (69). Mechanistically, blocking PD-1/PD-L1 interactions in these pCons-induced CD8^+^ Tregs reduced their Foxp3 expression and suppressive capacity, whereas the same blockade in naïve T cells increased Foxp3 (69). This indicates that PD-1 signaling can have divergent effects depending on the differentiation state of the T cell (53). The pCons-induced CD8^+^ Tregs exhibit a distinct phenotype characterized by low PD-1 expression, which is associated with their potent suppressive function (170). Conversely, in the NZB/WF1 model, another study found that CD4^+^PD-1^hi^ T cells infiltrating the kidney were proinflammatory, IFN-γ-producing cells, and treatment with an anti-PD-1 monoclonal antibody (mAb) reduced their numbers and alleviated nephritis, whereas anti-PD-L1 mAb exacerbated disease (171). This underscores the model- and subset-specific roles of the pathway.

The translation of these findings into human SLE therapy has been tested. Dapirolizumab pegol (DZP), a PEGylated anti-CD40L Fab’ fragment, was evaluated in a phase II trial. Although the primary endpoint based on BICLA responder rates at week 24 did not meet pre-specified dose-response criteria, all DZP groups showed improvements across multiple clinical and immunological measures compared to placebo, with a favorable safety profile (172). Following treatment withdrawal, disease activity scores stabilized but immunologic parameters returned toward baseline, suggesting a need for sustained therapy (172). This trial illustrates both the potential clinical benefit and the challenges of achieving robust, consistent responses with monotherapy targeting this complex immune network.

Given the limited success of single-agent checkpoint modulation and the multifaceted immune dysregulation in SLE, combination strategies targeting parallel or synergistic pathways are a major focus. The synergistic role of PD-1 with other inhibitory checkpoints like LAG-3 in preventing autoimmunity is evident from mouse models where dual deficiency precipitates lethal autoimmunity (173, 174). In the BXSB lupus model, a combined strategy of delivering the PD-1 inhibitory signal via adenoviral PD-L1 (Ad.PD-L1) while blocking the ICOS co-stimulatory pathway with anti-ICOSL mAb dramatically suppressed LN, outperforming either approach alone (175). This rationale is advanced by agents like acazicolcept (ALPN-101), a dual ICOS/CD28 antagonist, which demonstrated efficacy in reducing dermal and pulmonary fibrosis in systemic sclerosis models (176). Similarly, a bispecific inhibitor targeting both ICOSL and BAFF (AMG 570) was more efficacious than single inhibition in NZB/NZW lupus and arthritis models (177). These approaches aim to simultaneously restrain aberrant T cell help and B cell survival. Combining PD-1/PD-L1 axis modulation with agents that expand regulatory compartments is another promising avenue. Low-dose IL-2 therapy, which can promote Treg expansion, was shown to restore the Tfr/Tfh cell balance in SLE patients, and its efficacy was associated with this immunomodulatory effect (51). In murine lupus, nanoparticle-based delivery of IL-2 and TGFβ targeted to T cells via anti-CD2/CD4 antibodies expanded both CD4^+^ and CD8^+^ Tregs and suppressed disease (72). Furthermore, the calcineurin inhibitor tacrolimus, when combined with a STAT3 inhibitor, was shown to promote Treg populations while suppressing GC B cells and plasma cells *in vitro* (178), and its efficacy in a lupus model was synergistically enhanced by supplementation with *Lactobacillus acidophilus*, which modulated the Th17/Treg balance via pathways involving PD-L1 (121).

Cell-based therapies, particularly MSCs, have emerged as a potent immunomodulatory strategy with links to the PD-1/PD-L1 axis. Multiple studies and meta-analyses indicate that MSCs can ameliorate lupus symptoms and nephritis in both patients and murine models (116–118, 179). Their mechanism involves suppressing hyperactive T and B cells. The immunomodulatory function of MSCs can be enhanced by priming; for instance, phorbol ester-primed hMSCs inhibited B cell function in a PD-L1-dependent manner and showed superior efficacy in treating MRL/lpr mice (93). In BWF1 mice, bone marrow-derived MSCs inhibited B cell activation in an IFN-γ-dependent manner, mediated through cell contact involving the PD-1/PD-L1 pathway (94). A novel trained MSC product, HXB-319, designed to target inflammatory pathways, significantly improved survival, halted kidney disease progression, and modulated immune subsets including CD4^+^ PD-L1^+^ cells in a pristane-induced lupus model (119). This highlights how MSC therapies may partly exert their effect by modulating checkpoint interactions.

Beyond direct PD-1/PD-L1 targeting, therapeutic strategies often converge on inhibiting the pathogenic cell types driven by this axis. Since Tfh cells and their signature cytokine IL-21 are critical for aberrant GC responses in SLE, they are prime targets. IL-21 blockade in lupus-prone mice reduced autoantibodies, GC B cells, and nephritis by uncoupling Tfh-B cell interactions (84). Corticosteroids, a mainstay of SLE treatment, exert part of their effect by inhibiting the proportions and function of circulating Tfh cells (38, 180). Targeting upstream epigenetic regulators of Tfh differentiation also holds promise. Inhibition of Ezh2, a histone methyltransferase upregulated in lupus T cells, ameliorated disease in MRL/lpr mice and suppressed Tfh-driven autoantibody production and GC formation in a lupus-like cGVHD model, suggesting Ezh2 inhibitors could be repurposed for SLE (181, 182).

The experience with ICIs in oncology provides both caution and insight for SLE therapy. While ICIs like anti-PD-1 have revolutionized cancer treatment (183), they frequently induce irAEs, including rheumatic syndromes and, rarely, *de novo* SLE (184, 185). These irAEs underscore the physiologic role of checkpoints in maintaining self-tolerance. The phenomenon of hyperprogressive disease in some cancer patients treated with anti-PD-1 was linked to the expansion of highly suppressive PD-1^+^ effector Treg cells in tumors (186), mirroring the complex role of PD-1 on Tregs in lupus models (53). This highlights a critical consideration: systemic PD-1/PD-L1 blockade in autoimmunity risks exacerbating disease by removing inhibitory signals from exhausted effector cells or disrupting Treg function, whereas targeted agonism or combination strategies may offer a safer profile. Consequently, therapeutic development is shifting toward more precise interventions, such as targeting specific co-stimulatory pairs (e.g., ICOS/CD28), combining checkpoint modulation with cytokine therapy (e.g., IL-2), or using cellular therapies like MSCs that engage the PD-1/PD-L1 axis within a broader immunoregulatory program (94, 119).

**Figure 5:**
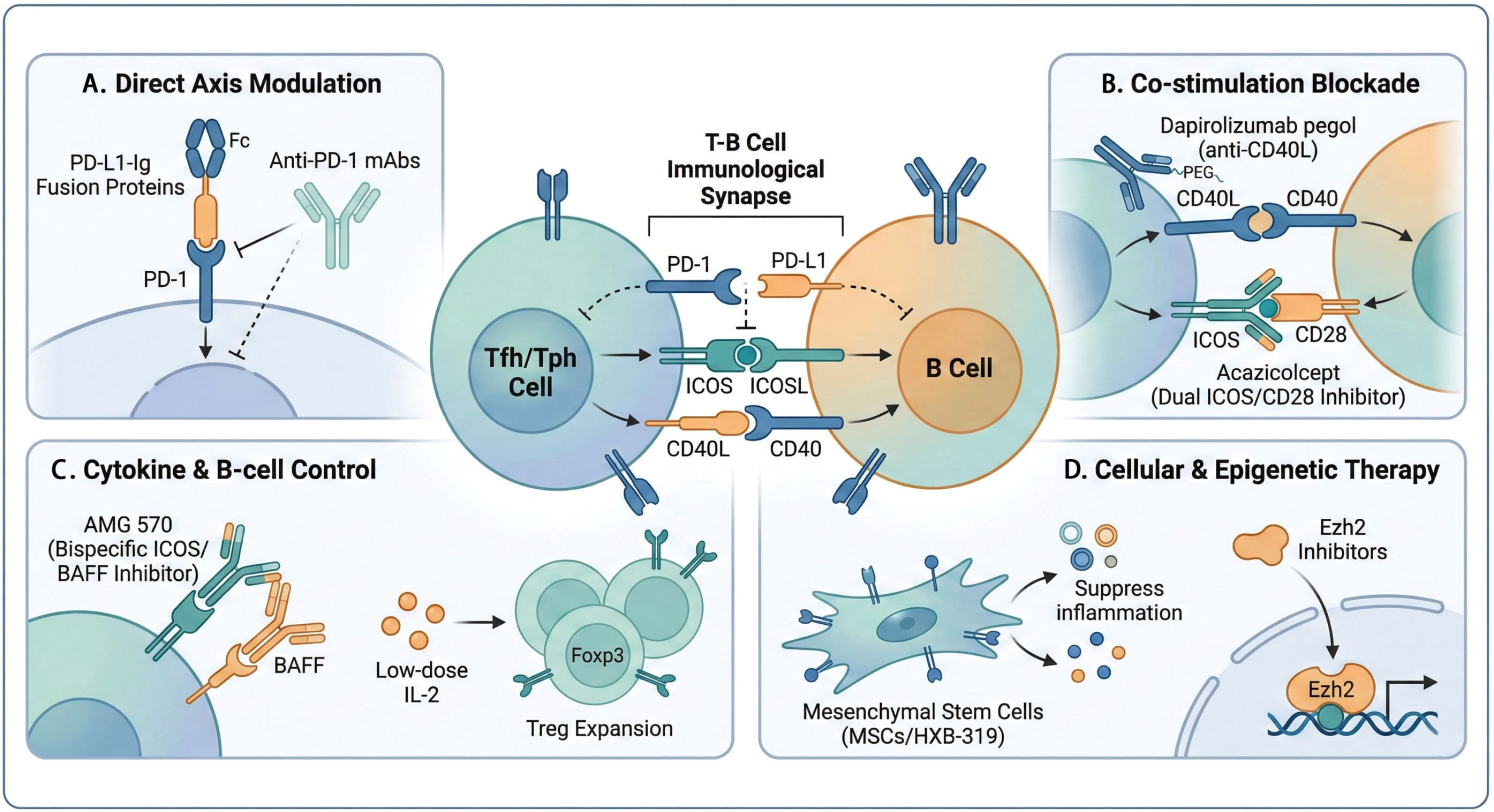
Precision immunotherapy strategies targeting the dysregulated PD-1 axis in SLE. **(A)** Direct Axis Modulation via PD-L1-Ig fusion proteins or agonistic anti-PD-1 mAbs. **(B)** Co-stimulation Blockade targeting CD40L (e.g., dapirolizumab pegol) or the ICOS/CD28 axis (e.g., acazicolcept). **(C)** Cytokine & B-cell Control utilizing bispecific inhibitors (e.g., AMG 570) and low-dose IL-2 to expand the Treg compartment. **(D)** Cellular & Epigenetic Therapy involving engineered MSCs (HXB-319) and Ezh2 inhibitors. BAFF, B-cell activating factor; Ezh2, enhancer of zeste homolog 2; ICOS, inducible T-cell costimulator; mAb, monoclonal antibody. Created with BioRender.com..

In summary, therapeutic targeting of the PD-1/PD-L1 axis in SLE is moving beyond simple checkpoint blockade toward sophisticated combinatorial and immunomodulatory strategies. Preclinical models demonstrate that both agonistic and antagonistic manipulations can be beneficial depending on context, highlighting the pathway’s duality. Clinical trials with agents like DZP show signals of efficacy but also underscore the challenges (172). The future lies in rationally designed combinations that simultaneously disrupt pathogenic Tfh-B cell axes (e.g., via ICOS/BAFF inhibition) (177), bolster regulatory networks (e.g., via low-dose IL-2 or Treg-inducing therapies) (51, 72), and leverage broad-spectrum modulators like engineered MSCs (119), all while carefully considering the lessons learned from onco-immunology to avoid precipitating or exacerbating autoimmunity (**Table 1**) (**Figure 5**). The emergence of B-cell-directed Chimeric antigen receptor T-cell (CAR-T) therapies, targeting CD19 or BCMA/BAFF, has demonstrated profound clinical efficacy in refractory SLE, raising the question of the continued relevance of checkpoint-based strategies. While CAR-T therapy offers a potent ablation of the B-cell compartment, the PD-1/PD-L1 axis remains a critical target for a more precise ‘recalibration’ of immune homeostasis. Unlike the systemic depletion of B cells, which can lead to prolonged hypogammaglobulinemia and infection risks, therapeutic agonists of the PD-1 pathway aim to restore the inhibitory ‘brake’ on hyperactive Tfh and Tph cells. This targeted restoration of self-tolerance addresses the upstream dysregulation of T-B cell crosstalk that characterizes SLE pathogenesis. Furthermore, given the significant cost and toxicity profile of current CAR-T protocols, checkpoint-modulating biologicals offer a more scalable and manageable alternative for the long-term maintenance of remission in a broader, heterogeneous patient population.

**Table 1:** Summary of clinical trials investigating PD-1/PD-L1 axis modulators and related immune checkpoint therapies in SLE.

Drug/Therapy name	Mechanism of action (Related to PD-1 axis)	NCT number/Status	Target population	Key evidence/Study highlights	References
DZP	PEGylated anti-CD40L Fab’ fragment; restores checkpoint balance indirectly	NCT04221451 (PHOENYCS)/Phase III	Moderate-to-severe active SLE	Phase II trials showed clinical and immunological improvements with a favorable safety profile	(172)
Acazicolcept (ALPN-101)	Dual ICOS/CD28 antagonist; blocks co-stimulatory signals	NCT04835441 (Synergy)/Phase II	Moderate-to-severe SLE	Demonstrated efficacy in preclinical models; currently in clinical evaluation for active SLE	(176)
AMG 570 (Rozibafusp Alfa)	Bispecific inhibitor of ICOSL and BAFF; targets T-B cell axis	NCT02983695/Phase IIb Completed	SLE (Refractory to Standard of Care)	Preclinical models showed superior efficacy over single blockade in lupus models	(177)
Low-dose IL-2 (Aldesleukin)	Expands Treg/Tfr cells to restore Tfr/Tfh balance	NCT02411214 (Example)/Phase II	Active SLE	Multicenter RCT studies show increased SRI-4 response and reduced anti-dsDNA antibodies.	(51)
HXB-319 (Engineered MSC)	“Trained” MSCs; induces apoptosis via PD-1/PD-L1 pathway	Preclinical/Early Clinical	LN	2025 studies confirmed efficacy in controlling progressive glomerulonephritis and proteinuria	(119)
PD-L1-Ig Fusion Protein	Directly activates PD-1 inhibitory signaling	Preclinical Research	SLE/Nephritis (BWF1 model)	Significantly prolonged survival and alleviated nephritis symptoms in murine models	(169)
Ezh2 Inhibitor	Targets epigenetic regulators of Tfh differentiation	Preclinical Research	SLE (MRL/lpr models)	Ameliorated lupus-like disease and autoantibody production in preclinical studies	(181, 182)

## 9 Conclusions and future perspectives

The fundamental paradox of the PD-1/PD-L1 axis in SLE—serving as both a critical brake on autoreactivity and a paradoxical facilitator of humoral autoimmunity under specific cellular and microenvironmental contexts—encapsulates the profound complexity of this disease. This duality underscores a central tenet of modern lupus immunology: systemic autoimmunity arises from a coordinated breakdown across multiple layers of immune regulation, where the same molecular pathway can exert divergent effects depending on the cellular subset, differentiation state, and inflammatory milieu (1, 3, 10). The variable outcomes in clinical trials, as seen with agents like DZP (172), are not failures of the target per se but reflections of our incomplete mapping of this immunological terrain in a heterogenous patient population. Resolving this paradox for therapeutic gain, therefore, necessitates moving beyond a one-size-fits-all approach toward a precision immunotherapy framework that is predicated on patient stratification, pathway context, and combinatorial logic.

The extreme heterogeneity of SLE, manifested in over 180 different autoantibodies (187) and driven by diverse genetic, epigenetic, and environmental interactomes (1, 188), remains the primary challenge in clinical translation. This heterogeneity extends to the very pathways of pathogenic B cell generation, with individual patients likely exhibiting dominance of either GC or extrafollicular plasma cell differentiation routes, each with distinct cellular requirements and checkpoint dependencies (85). Similarly, epigenetic programming imprints a pathogenic signature on naïve B cells early in the disease course (80), which may dictate their subsequent responsiveness to co-stimulatory or co-inhibitory signals. Consequently, the net effect of modulating the PD-1/PD-L1 axis will depend on which pathogenic circuit is active, the pre-existing balance of Tfh and Tfr cells (88, 189), and the functional state of innate immune sentinels that express PD-L1. The lesson from dapirolizumab is instructive: while the primary endpoint was not met, improvements across multiple clinical and immunological measures were observed (172), suggesting a biological effect obscured by unrefined patient selection and outcome measures. Future efforts must identify biomarkers that can stratify patients based on their dominant immunopathogenic module to determine who might benefit from PD-1 pathway modulation.

The experience from oncology provides a crucial, albeit cautionary, roadmap. ICIs against PD-1/PD-L1 can induce *de novo* autoimmune phenomena, including lupus-like syndromes and other rheumatic immune-related adverse events (Rh-irAEs) (184, 190). These events highlight the physiological role of these checkpoints in maintaining peripheral tolerance and demonstrate that their blockade can unleash autoreactive lymphocytes (191). This mirror image of the intended therapeutic effect in cancer informs the risk profile for SLE therapy and emphasizes that any intervention must be finely calibrated to re-establish tolerance without causing global immunosuppression or triggering other autoimmune pathologies. Management strategies developed for Rh-irAEs, including the use of corticosteroids and conventional disease-modifying antirheumatic drugs (185, 191), may need to be integrated into treatment protocols for SLE patients receiving checkpoint-targeted therapies. Furthermore, the pursuit of combination therapies, which is central to enhancing efficacy in cancer immunotherapy (192, 193), is equally imperative in SLE. Rational combinations could simultaneously target multiple nodes of dysregulation; for example, a PD-1 pathway modulator might be combined with a BAFF blocker to constrain B cell survival (194), an anti-IL-21 agent to disrupt Tfh help (84, 189), or a low-dose IL-2 regimen to bolster regulatory T cell function (51).

The future of targeting the PD-1/PD-L1 axis in SLE likely lies not in standalone blockade or agonism, but in its integration into broader immunomodulatory strategies that reshape the immunological landscape. MSCs, with their multifaceted capacity to suppress effector responses, promote regulatory networks, and potentially upregulate PD-L1 on target cells, represent one such approach (116, 117, 179). Engineered cellular therapies or tolerogenic vaccines, such as the pCons peptide that induces regulatory CD8^+^ T cells with a distinct phenotype including low PD-1 expression (170), offer avenues to specifically correct the immune imbalance. Similarly, modulating the gut-immune axis, as shown with *Lactobacillus acidophilus* supplementation enhancing tacrolimus efficacy by influencing Th17/Treg balance and PD-L1 expression (121), opens another dimension for adjunctive therapy. The goal is to shift the system from a state of chronic activation and broken tolerance (195, 196) to one of controlled homeostasis, where the PD-1/PD-L1 axis can resume its physiological role as a gatekeeper of appropriate immune responses.

In conclusion, the path forward requires a synergistic convergence of deep phenotyping, biomarker discovery, and innovative trial design. We must leverage genomic, epigenomic (80), proteomic, and cellular profiling to define patient endotypes, distinguishing those with PD-1/PD-L1 dysfunction central to their pathology from those where other checkpoints like CD40/CD40L or ICOS/ICOSL are primary drivers (11). Furthermore, a promising yet under-explored area involves linking genomic variations within the PDCD1 locus directly to the quantitative frequency of specific T-cell subsets. Although it is recognized that the PD1.3A polymorphism correlates with reduced PD-1 expression, future investigations should prioritize defining how these genetic blueprints predispose individuals to the expansion of pathogenic helper subsets like Tfh or Tph cells. Establishing these genotype-phenotype correlations will be instrumental for advancing personalized treatment approaches. By integrating genetic risk scores with real-time immune profiling, clinicians could move toward a precision framework where interventions—such as checkpoint agonists or subset-specific modulators—are selected based on the patient’s dominant immunopathogenic module. Clinical trials must adopt composite endpoints that capture the multidimensional nature of SLE response (197) and may need to enroll patients based on molecular signatures rather than purely clinical criteria (198). Ultimately, taming lupus (2) will depend on our ability to decipher the unique “interactome” of each patient (1) and deploy a modular therapeutic arsenal that includes precision-targeted checkpoint modulators, not as magic bullets, but as strategically chosen components of a personalized immunologic reset. Resolving the PD-1/PD-L1 paradox is therefore not about finding a single answer, but about learning to ask the right questions for each individual living with this complex disease.

## 10 Literature search strategy and selection criteria

Data for this review were identified by searches of PubMed and Web of Science for articles. The search involved combinations of the following terms: “Systemic Lupus Erythematosus”, “PD-1”, “PD-L1”, “PD-1/PD-L1 signaling axis”, “B cell immunotolerance”, “Tph cells”, and “checkpoint agonists”.
